# Dissection of Splicing Regulation at an Endogenous Locus by Zinc-Finger Nuclease-Mediated Gene Editing

**DOI:** 10.1371/journal.pone.0016961

**Published:** 2011-02-08

**Authors:** Sandra Cristea, Philip D. Gregory, Fyodor D. Urnov, Gregory J. Cost

**Affiliations:** Sangamo BioSciences, Richmond, California, United States of America; Centre de Regulació Genòmica, Spain

## Abstract

Sequences governing RNA splicing are difficult to study *in situ* due to the great difficulty of traditional targeted mutagenesis. Zinc-finger nuclease (ZFN) technology allows for the rapid and efficient introduction of site-specific mutations into mammalian chromosomes. Using a ZFN pair along with a donor plasmid to manipulate the outcomes of DNA repair, we introduced several discrete, targeted mutations into the fourth intron of the endogenous *BAX* gene in Chinese hamster ovary cells. Putative lariat branch points, the polypyrimidine tract, and the splice acceptor site were targeted. We recovered numerous otherwise isogenic clones carrying the intended mutations and analyzed the effect of each on *BAX* pre-mRNA splicing. Mutation of one of three possible branch points, the polypyrimidine tract, and the splice acceptor site all caused exclusion of exon five from *BAX* mRNA. Interestingly, these exon-skipping mutations allowed usage of cryptic splice acceptor sites within intron four. These data demonstrate that ZFN-mediated gene editing is a highly effective tool for dissection of pre-mRNA splicing regulatory sequences in their endogenous context.

## Introduction

The use of mutagenesis to reveal gene function is a classic technique in biology. The difficulty of achieving targeted mutagenesis in mammalian cells often has necessitated the use of extra-chromosomal or randomly-integrated reporter constructs as a proxy for endogenous gene function. While reporter-based experiments have contributed immensely to our understanding of the cell, loss of correct gene dosage, regulation, and chromatin structure can misrepresent the biology of the endogenous gene. In the case of RNA splicing, use of reporter genes can be unusually problematic as splicing is influenced or regulated by large-scale processes like chromatin modification [1], [2], [3], [4], [5], transcription [6], [7], , and mRNA export [13], [14]. Furthermore, the large size of mammalian genes often simply precludes the use of reporter systems to analyze splicing. Retrospective analysis of splicing in cells with naturally-occurring mutations has been informative but is not compatible with directed experimentation and lacks appropriate isogenic controls [15], [16], [17]. Given the centrality of alternative splicing to metazoan biology and disease, techniques that allow investigation of splicing regulation in its natural context are sorely needed.

**Figure 1:**
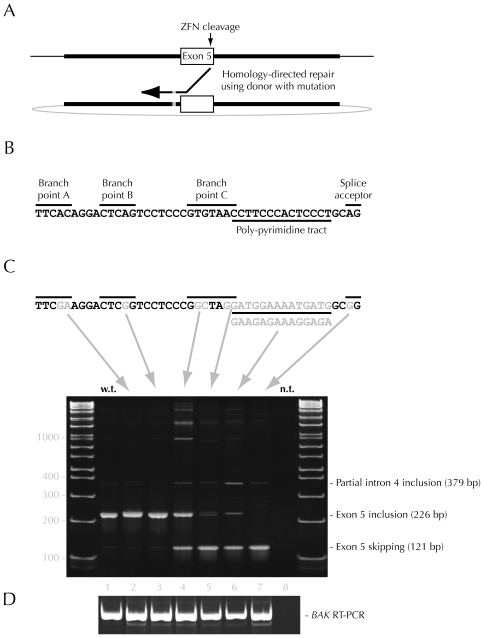
Alteration of *BAX* pre-mRNA splicing in response to mutagenesis of key *cis* regulatory splicing sequences. A) Diagram of homology-directed repair in the *BAX* exon five region. Cleavage at the end of exon five stimulates strand invasion by the resected single-strand DNA. Once base pairing is established between the chromosome and the donor, new DNA synthesis and repair of the break using the newly synthesized DNA results in incorporation of the mutated sequence into the chromosome. Thick black lines, homology between the chromosome (top) and the donor plasmid (bottom); black arrow, strand invasion and new DNA synthesis; grey patch in donor plasmid and new DNA, mutation. Exon five and the donor sequence are drawn to scale. B) Putative splice site sequences found within the 3′ end of *BAX* intron four. Potential lariat branch points, the polypyrimidine tract, and the splice acceptor site in *BAX* intron four are indicated with black bars. The site of ZFN cleavage (shown in grey) is approximately 90 bp from the end of exon five. C) Mutations introduced into *BAX* intron four and their effect on *BAX* splicing. The location of specific base changes made in each isogenic cell line are shown in grey. For the branch point C mutation, the terminal C of the consensus sequence shown in part A was not altered. Arrows link the mutation with the gel lane containing RT-PCR products from a cell clone bearing the corresponding mutation. Lane 1; wild-type CHO-K1 cells; lane 2, branch point A mutation; lane 3, branch point B mutation; lane 4, branch point C mutation; lane 5, mutation of branch point C and the polypyrimidine tract (top); lane 6, mutation of the polypyrimidine tract (bottom); lane 7, splice acceptor mutation; lane 8, no template control. The identity and size of bands excised and confirmed by Sanger sequencing is shown to the right of the gel. High-molecular weight bands present in lanes 4–7 are heteroduplex material formed late in the PCR reaction by annealing of normal and exon five-skipped RT-PCR products. Molecular weight markers are in base pairs. The splicing pattern for each mutation was assayed between 4 and 12 times; representative data are shown. D) RT-PCR of the wild-type *BAK* gene in all eight samples serves as a loading control and is shown below the *BAX* RT-PCR.

A ZFN pair creates a targeted double-strand break in chromosomal DNA. When repaired inaccurately by the non-homologous end joining (NHEJ) DNA repair machinery, gene disruption results [18], [19], [20], [21]. Alternately, the homology-directed DNA repair (HDR) pathway can be manipulated to engineer mutations into endogenous genomic loci [22], [23], [24], [25]. In this application, a plasmid with chromosomal DNA sequence flanking the ZFN cleavage site and containing the desired mutation is co-delivered with the ZFNs. The cell can use this donor molecule as a template for DNA repair, resulting in copying of the mutated region into the chromosome (Figure 1A).

The *BAX* gene is haploid in CHO cells and its mutation by NHEJ can result in loss of a splice acceptor site resulting in skipping of exon five [26]. We reasoned that engineered mutagenesis of the endogenous gene via HDR would allow us to dissect the sequences required for normal *BAX* splicing. Here we demonstrate the utility of ZFNs to reveal splice site sequences. We introduce six mutations into intron four of the endogenous *BAX* gene and assay their effect on splicing by RT-PCR and high-throughput sequencing. Notably, our targeted mutagenesis identifies the branch point nucleotide for intron four lariat formation and uncovers cryptic splice acceptor regions within intron four, providing the first *in situ* functional annotation of splice site sequences in mammalian cells.

## Methods

### Donor plasmid design and construction

Donor plasmids were created with *BAX* homology regions on either side of the targeted mutations using fusion PCR. Each homology arm was synthesized separately by PCR amplification of *BAX* genomic DNA with a distal primer and a central primer containing the targeted mutation. For the initial round of PCR, the left homology arms were synthesized using GJC 47F (5′-cag aag ttc aag att ggc tta c-3′) and a mutation-specific reverse primer while right homology arms were created with a partially-complementary mutation-specific forward primer and GJC 114R (5′-tga acc agg ctg gga gat tt -3′). Annealing of the complementary ends containing the mutated regions during a second round of PCR created a 882 bp region of homology with a central intronic mutation. This second PCR was done using two primers inside GJC 47F and GJC 114R, namely GJC 250F (5′-cca gcc tgg ttt aca cag at-3′) and GJC 249R (5′-ggt tct gca taa gtg gtt ct-3′). Each resulting amplicon was cloned into the pCR2.1 vector (Invitrogen) and clones confirmed by DNA sequencing. All PCR amplifications were done with Accuprime Taq Hi Fidelity DNA Polymerase (Invitrogen) per the manufacturer's recommendations.

Mutation-specific oligonucleotides are shown below with mutations in bold. For the branch point A donor, the left homology arm was created by PCR of the *BAX* locus with GJC 47F and SC 12R #3 (5′-agt cct **tc**g aac act cct gga gaa ggt ggt-3′); **the right homology arm by PCR with SC 13F #3 (5′-agt gtt c**ga** agg act cag tcc tcc cgt gt-3′**) and GJC 114R. The branch point B donor was made as above but using SC 12R (5′-ggg agg ac**c** gag tcc tgt gaa cac tcc tgg-3′ **and SC 13F (5′-gga ctc **g**gt cct ccc gtg taa cct tcc ca-3′**). The branch point C donor was made as above but using SC 12R #2 (5′-ag**g** **c**ta **g**cc ggg agg act gag tcc tgt gaa-3′ **)** **and SC 13F #2 (5′-cct ccc gg**c** ta**g** **c**ct tcc cac tcc ctg cag-3′**). The pY tract donor with AG dinucleotides was made as above but using SC 14R (5′-**gct ctc ctt tct ctt c**tt aca cgg gag gac tga g-3′ **)** **and SC 15F (5′- **gaa gag aaa gga gag c**ag gcc ctg tgt acc aaa g -3′**). The pY tract donor with no AG dinucleotides was made as above but with SC 14_2R (5′-t**gc cat cat ttt cca tc**t tac acg gga gga ctg a-3′) **and SC 15_2F (5′-**gat gga aaa tga tgg c**ag gcc ctg tgt acc aaa g-3′**). The splice acceptor donor was made as above but with SC 16R (5′- cag ggc **cc**g cag gga gtg gga agg tta-3′ **and SC 17F (5′- tcc ctg c**gg** gcc ctg tgt acc aaa gtg-3′**).

### Transfection and cell cloning

CHO-K1 cells were obtained from the American Type Culture Collection (ATCC CCL-61) and grown and transfected with the FokI EL/KK heterodimer mutant *BAX* ZFNs as described previously (Figure S1) [26]. Twenty micrograms of donor was cotransfected with the ZFNs. Genomic DNA of the transfected pools was extracted from the aforementioned samples using the Masterpure kit (Epicentre). Each sample was PCR-amplified using GJC 47F and GJC 114R, oligonucleotides outside of the donor sequence. This PCR was used to analyze *BAX* ZFN action via the Cel I assay and to analyze the extent of targeted mutation by assay of restriction fragment length polymorphism (RFLP). For the Cel I assay, a nested PCR was performed with a primer closer to the ZFN sites, GJC 52F (5′- cag agg aat gaa agc aaa gg-3′), and GJC 114R. The percentage of modified cells was calculated as described previously [19], [26]. For the RFLP assay, each donor was designed so that the targeted mutation either deletes or creates a new restriction site relative to the unmodified sequence. The branch point A mutation creates a BstBI site; the branch point B mutation creates a Sau961 site; the branch point C mutation creates an NheI site; the two polyY tract mutations and the splice acceptor mutation delete a PstI site. Digestion with the corresponding diagnostic restriction enzyme indicated the extent of specific modification by HDR.

For cell cloning, each co-transfected pool was diluted to 0.4 cells/well in 96-well plates. After approximately 2 weeks of growth, samples from each occupied well were harvested and genomic DNA isolated using the Epicentre QuickExtract Solution. Each sample was PCR amplified with GJC 47F and GJC 114R and analyzed by the RFLP assay described above. Positive clones were expanded and then genotyped by DNA sequencing.

### RNA analysis

Total RNA was prepared from each clone with the High Pure RNA Isolation Kit (Roche). Complementary DNA was synthesized using a polydT oligonucleotide to prime reverse transcription by the SuperScript III RT (Invitrogen). For analysis of *BAX*, the cDNA from each clone was PCR amplified using GJC 12F (in exon four, 5′-ctt ctt ccg tgt ggc agc tg-3′) and GJC 23R (in exon six, 5′-ccc gaa gta tga gag gag gcc atc -3′), and PCR analyzed by electrophoresis on 10% polyacrylamide gels. The identity of each *BAX* cDNA band annotated in Figure 1 was confirmed by Sanger DNA sequencing. For analysis of *BAK*, cDNA was PCR amplified using GJC164F (in exon 1, 5′-cgg gat ctt tgt ctt cag ac-3′) and GJC 25R (in exon 4, 5′-ctg gaa ctc tgt gtc gta tct ccg g-3′).

### High-throughput DNA sequencing

Solexa/Illumina-based DNA sequencing was performed on a pool of RT-PCR products from the clone in lane six (the polypyrimidine tract mutation containing AG dinucleotides). cDNA from this clone was PCR amplified with a forward oligonucleotide in exon four (5′- aat gat acg gcg acc acc gag atc tac act ctt tcc cta cac gac gct ctt ccg at**c tag caa act ggt gct caa g**-3′) **and a reverse oligonucleotide in exon 6 (5′-caa gca gaa gac ggc ata cga gct ctt ccg atc t**aa gta tga gag gag gcc atc**-3′**) where bold font indicates homology to *BAX* genomic DNA. The amplicon was purified first with the Qiaquick PCR Purification kit (Qiagen) then with the GeneJET Purification Protocol (Fermentas).

## Results

The splicing machinery recognizes several key splice site sequences in pre-mRNA to effect intron removal. In brief, binding of U2AF to the polypyrimidine tract promotes formation of a 5′-2′ lariat linkage between the splice donor site and the branch point nucleotide. The resulting lariat molecule is excised by nucleophilic attack of the splice acceptor site by the upstream exon. Potential lariat branch points, polypyrimidine tracts, and splice acceptor sites in *BAX* intron four were identified computationally by the Sroogle algorithm and by visual inspection (Figure 1B) [27], [28], [29], [30]. Six donor plasmids were assembled, each containing 882 bp of flanking BAX genomic DNA along with six separate mutated regions designed to disrupt each of three potential lariat branch points, the polypyrimidine tract (two mutations), and the splice acceptor site, respectively. *BAX* ZFNs were transfected into CHO-K1 cells with each individual donor plasmid. Mutation at *BAX* three days post-transfection was 6.3+/−2.0% (n = 6) of alleles as measured by the Cel I assay [19]. The transfected pool was cloned by limiting dilution and *BAX* PCR products from each clone screened for the gain or loss of a restriction site generated by the intended mutation in *BAX* intron four. The genotype of positive clones was confirmed by DNA sequencing. Across all six transfections, correct clones were recovered at a frequency of 0.3+/−0.2% (n = 6; see Discussion).

RNA was prepared from wild-type CHO-K1 cells and from one representative clone for each of the six mutations. RT-PCR was performed using primers in exons four and six. Analysis of PCR products for each mutation revealed the contribution of each sequence to the correct splicing of exon five (Figure 1C). Firstly, mutation of putative branch points A and B did not affect splicing. Both mutants resulted in a single RT-PCR band indistinguishable from that in wild-type cells, demonstrating that genome editing *per se* does not alter RNA splicing. In contrast, loss of branch point C caused an approximate 50% loss of exon five from the BAX transcript (lane 4). Both mutations of the polypyrimidine tract resulted in very significant loss of correct splicing (∼90%, lanes 5,6). As expected, a single nucleotide change in the highly-conserved splice acceptor site completely prevented splicing of exon five (lane 7). The level of *BAK* mRNA (an unmodified, wild-type gene) was unaffected by any of the mutations introduced into *BAX* (Figure 1D).

Certain mutations caused the appearance of or an increase in of minor splice forms (lanes 4–7). To sample splice form diversity, we subjected RT-PCR products from lane six to high-throughput DNA sequencing using a forward primer placed at the very end of exon four. Five different transcripts containing varying lengths of the 3′ end of intron four were found: inclusion of 153, 50, 38, 142, and 31 bp, in order of approximate frequency. All such cryptic splice sites are immediately preceded by an YAG splice acceptor sequence. Clearly visible in lanes 4–7, the most abundant cryptic splice site (379 bp band) has a well-positioned YUNAY branch point consensus and polypyrimidine tract (cttac-ctcttccctgccttcc-ag). None of the mRNAs derived from cryptic splicing result in a 5′ extension of the normal *BAX* exon 5 open reading frame; all transcripts except the rarest 31 bp extension should be degraded by nonsense-mediated mRNA decay.

Taken together, these data demonstrate the successful creation of isogenic CHO cells carrying an allelic series of mutations implicated in the regulation of splicing at the endogenous *BAX* gene. Our data reveal the critical importance of just one of three potential branch points, the polypyrimidine tract and the splice acceptor sequence in the maintenance of normal exon five splicing. Moreover, these data reveal cryptic splice sites in intron four active only when the preferred natural site is mutated.

## Discussion

Experiments done *in vitro* and in yeast have informed our knowledge of splicing biology enormously, yet understanding has been hampered in most organisms by a simple inability to perform targeted mutagenesis. Such a facility would be particularly useful for understanding the integration of splicing with chromatin remodeling and transcription and for studying the alternative splicing that accounts for a large part of biological diversity in higher eukaryotes. We performed ZFN-mediated targeted mutagenesis of the *C. griseus BAX* gene to dissect the importance of various splice site sequences in their normal, endogenous chromosomal context. Traditional recombination is also capable of creating site-specific mutations in endogenous genes but is laborious and time-consuming. In contrast, the mutations described here were created in two overlapping tranches, each of ∼10 weeks in duration, from donor creation to isolation of the final clones. Most of this period involved clone growth and expansion and therefore little investigator effort. The inefficiency of traditional homologous recombination necessitates co-integration of a selectable marker gene. The ability to obtain the desired mutations without the use of a selectable marker obviates concerns about the confounding impact of such an exogenous, independent transcription unit.

The conversion efficiency of double-strand breaks into correctly mutated alleles in this experiment was much lower than the amount of ZFN cleavage (6.3% cleavage, 0.3% conversion) and lower than the ∼33% of breaks typically converted into targeted integrations [25], (data not shown). This is likely the result of the ∼110 bp between the site of the ZFN-induced break and the site of the intended mutations. Because of this distance, the break can be repaired successfully without single-strand extension long enough to reach the mutated site in the donor plasmid. ZFNs can be targeted to essentially any DNA sequence. Use of a custom ZFN pair positioned more closely to the target area should substantially improve the relative frequency of mutated clones. Furthermore, only 3′ end invasion from the right side of the break will result in the introduction of the mutation. (Invasion from the left will copy only wild-type sequence from the donor plasmid.) Despite the modest overall efficiency of mutagenesis, we were still able to isolate the desired mutants in a single round of experimentation.

*BAX* is naturally haploid in CHO cells. Exon five is in frame with exon four and the terminal exon six; transcripts lacking exon five will therefore not be subject to nonsense-mediated mRNA decay and will provide an unbiased readout of *BAX* splicing. Interrogation of most other genes will require a similar choice of an in-frame exon and the generation of homozygous mutant cells. Homozygotes are typically formed at a approximately one-third the frequency of heterozygous mutants or can be made via a second round of ZFN mutagenesis [23]. Alternately, the complications of diploidy can be avoided by use of a near-haploid cell line such as the human leukemia line P1-55 [31].

Mutation of branch point consensus C resulted in an incompletely penetrant splicing phenotype. We suspect that an second lariat branch point was used to produce the correctly-spliced fraction of *BAX* mRNA, perhaps sites A or B. Similar usage of a back-up, cryptic branch point sequence has been seen in the case of the β-globin gene [32]. Alternately, perhaps the lariat is simply formed at the mutated site with lower efficiency [33]. Creation of a doubly-mutant allele will be needed to identify the back-up branch point conclusively.

Candidate branch point C was not identified in the initial screen of intron four for splicing-related sequences. As a result, the mutations made to the polypyrimidine tract overlap the branch point C consensus sequence by one nucleotide. The splicing defect seen in lanes five and six and might therefore reflect loss of both polypyrimidine tract and branch point C function. As loss of branch point C function alone (lane 4) results in only partial exclusion of exon five from *BAX* mRNA, the near total skipping of exon five seen with these potentially compound mutants indicates a significant contribution of the polypyrimidine tract to correct *BAX* splicing.

We made two mutations to the polypyrimidine tract. The second mutation attempted to alter splice acceptor site usage by inclusion of multiple AG dinucleotides (lane six). In this context, the lariat branch point sequence could be provided by the A or B sites and the polypyrimidine tract by the run of seven Ys between branch points B and C. (Seven pyrimidines are sufficient for U2AF binding [34].) We did not observe usage of these potential splice acceptor sites even by high-throughput DNA sequencing. As the primary purpose of this mutation was to disrupt the polypyrimidine tract, we did not include the pyrimidine typically present just 3′ of a strong splice acceptor site. This omission likely accounts for the failure of these AG dinucleotides to be used as cryptic splice sites. All five of the cryptic splice sites found by high-throughput DNA sequencing have a YAG splice acceptor site, whereas only the most abundant has a readily-identifiable lariat branch point and polypyrimidine tract.

## Supporting Information

Figure S1 **The full nucleotide sequence of the** ***BAX*** **ZFN expression plasmid.**
(TXT)
